# Supplementary material for: Is amblyopia associated with school readiness and cognitive performance during early schooling? Findings from the Millennium Cohort Study
Source: PLoS One. 2020 Jun 19;15(6):e0234414. doi: 10.1371/journal.pone.0234414 (PMC7304573; doi:10.1371/journal.pone.0234414)
Supplement: S1 Table — (DOCX) [file pone.0234414.s001.docx]

Table S1: Coding of covariates.

| Covariate | Description | Coding |
| --- | --- | --- |
| Amblyopia and/or strabismus status | Case definition: at least one parental report of amblyopia and/or strabismus and at least one report on related treatment at survey sweeps 2-4 (child aged 3, 5, or 7). | No eye condition (reference) / refractive amblyopia / strabismic or mixed amblyopia / strabismus alone. |
| BAS II NV | Outcome: British Ability Scale II Naming Vocabulary. This test assesses expressive language ability and long-term memory. Age-standardised scores were used with mean 50 and standard deviation 10. The test was measured at the second and third survey sweeps (child aged 3 and 5 years). | Continuous |
| BAS II PC | Outcome: British Ability Scale II Pattern Construction. This test assesses spatial awareness, visual perception, planning and decision making. Age-standardised scores were used with mean 50 and standard deviation 10. The test was measured at the second and third survey sweeps (child aged 3 and 5 years). | Continuous |
| Birth order | The birth order of the child, measured at first survey sweep (child aged 9 months). | 1 (reference) / 2 / 3+. |
| BSRA-R | Outcome: Bracken School Readiness Assessment. This test assesses educationally relevant concepts needed for early formal education. Poor school readiness was defined as scoring in the bottom 25 percentiles. The test was measured at the second survey sweep (child aged 3 years). | No (reference) / yes |
| Ethnicity | Ethnicity of child, measured at first survey sweep (child aged 9 months). | White (reference) / non-white. |
| Eye conditions | Any visual impairment or blindness due to any cause including cerebral visual impairment and the following ocular conditions: nystagmus, ptosis, anophthalmos, glaucoma, anterior segment abnormality, craniofacial disorders, corneal opacity and dystrophy, cataract, retinal detachment, retinal dystrophy, albinism, retinopathy of prematurity, retinal haemorrhage, retinal coloboma, retinitis, coloboma, aniridia, uveal tumour, and uveitis, optic nerve hypoplasia, secondary atrophy, optic neuropathy/neuritis, optic nerve tumour, coloboma, and ocular trauma to the eye. Children with these eye conditions were excluded from the analyses. | No (reference) / yes. |
| Gestational age | Child born before 37 weeks of gestational age, measured at first survey sweep (child aged 9 months). | No (reference) / yes. |
| Income | Annual disposable household income, measured for 95% at first survey sweep and 5% at second survey sweep (child aged 9 months or 3 years). | ≥£20800 (reference) / £10400-£20800 / <£10400. |
| Language | Main language spoken at home, measured at first survey sweep (child aged 9 months). | English (reference) / non-English. |
| Maternal education | Highest obtained qualification by mother of child, measured at first survey sweep (child aged 9 months). | Higher degree, diploma, or A-levels (reference) / O-levels or other / no qualifications. |
| Neurological conditions | All ICD10 G-codes except for G43 migraine, G44 headache, and G47 sleep disorder. Children with neurological conditions were excluded from the analyses. | No (reference) / yes. |
| Sex | Sex of child, measured at first survey sweep (child aged 9 months). | Girl (reference) / boy. |
| Treatment | At least one parental report that child had eye surgery, occlusion by patch or penalisation using cycloplegic drops, and/or spectacles, measured at survey sweeps 2-4 (child aged 3, 5, or 7). | For each treatment type: No (references) / yes. Combined started treatment, time-variant: No (reference) / age 3 / age 5 / age 7. |
