# Supplementary material for: Is amblyopia associated with school readiness and cognitive performance during early schooling? Findings from the Millennium Cohort Study
Source: PLoS One. 2020 Jun 19;15(6):e0234414. doi: 10.1371/journal.pone.0234414 (PMC7304573; doi:10.1371/journal.pone.0234414)
Supplement: S2 Table — a Missing data in confounders (n = 471): gestational age (n<10), maternal education (n = 32), and disposable household income (n = 445). b Missing data cognitive tests (based on the number of children participating at the oldest age at which the cognitive test was taken): Bracken School Readiness Assessment-Revised (n = 1460), British Ability Scale II Naming Vocabulary at ages 3 and 5 (n = 774), and British Ability Scale II Pattern Construction at ages 5 and 7 (n = 313). c Odds ratios adjusted for all covariates listed in the table and sample weights. (DOCX) [file pone.0234414.s002.docx]

Table S2: Odds of missing data in confounders and cognitive tests.

| Covariate | Category | Confounders  OR (95%CI) | BSRA-R age 3  OR (95%CI) | BAS II NV ages 3-5  OR (95%CI) | BAS II PC ages 5-7  OR (95%CI) |
| --- | --- | --- | --- | --- | --- |
| BSRA-R age 3 | | 1.00 (0.99-1.02) | NA | NA | NA |
| BAS II NV age 3 | | 1.01 (0.99-1.02) | NA | NA | NA |
| BAS II NV age 5 | | 0.99 (0.97-1.01) | NA | NA | NA |
| BAS II PC age 5 | | 1.00 (0.98-1.02) | NA | NA | NA |
| BAS II PC age 7 | | 1.01 (0.99-1.03) | NA | NA | NA |
| Eye condition | No eye condition | 1.00 | 1.00 | 1.00 | 1.00 |
|  | Refractive amblyopia | 1.28 (0.31-5.23) | 1.39 (0.84-2.30) | 0.96 (0.46-2.01) | 1.08 (0.52-2.24) |
|  | Strabismic or  mixed amblyopia | 0.10 (0.01-0.74) | 1.09 (0.51-2.34) | 0.98 (0.20-4.86) | 0.85 (0.31-2.12) |
|  | Strabismus alone | 0.30 (0.07-1.21) | 0.83 (0.51-1.34) | 1.06 (0.57-1.97) | 1.70 (0.97-2.96) |
| Sex | Girls | 1.00 | 1.00 | 1.00 | 1.00 |
|  | Boys | 0.85 (0.62-1.16) | 1.52 (1.33-1.73) | 1.47 (1.23-1.75) | 1.41 (1.18-1.70) |
| Ethnicity | White | 1.00 | 1.00 | 1.00 | 1.00 |
|  | Non-white | 1.21 (2.74-6.46) | 2.82 (2.38-3.33) | 2.76 (2.21-3.44) | 1.43 (1.09-1.89) |
| Birth order | 1 | 1.00 | 1.00 | 1.00 | 1.00 |
|  | 2 | 1.25 (0.86-1.81) | 0.97 (0.84-1.13) | 1.15 (0.94-1.42) | 1.01 (0.81-1.25) |
|  | 3+ | 1.79 (1.21-2.63) | 1.02 (0.86-1.21) | 1.25 (1.01-1.56) | 1.09 (0.86-1.39) |
| Household language | English | 1.00 | 1.00 | 1.00 | 1.00 |
|  | Non-English | 2.82 (1.50-5.30) | 1.45 (1.07-1.95) | 2.21 (1.55-3.14) | 0.92 (0.55-1.53) |
| Gestational age | ≥37 weeks | NA | 1.00 | 1.00 | 1.00 |
|  | <37 weeks | NA | 1.13 (0.89-1.43) | 0.91 (0.64-1.29) | 0.97 (0.69-1.37) |
| Maternal education | A-levels or higher | NA | 1.00 | 1.00 | 1.00 |
|  | O-levels | NA | 1.16 (0.99-1.36) | 1.29 (1.03-1.63) | 1.42 (1.12-1.81) |
|  | None | NA | 1.55 (1.26-1.91) | 2.14 (1.63-2.80) | 1.98 (1.47-2.66) |
| Household income | ≥£20800 | NA | 1.00 | 1.00 | 1.00 |
|  | £10400-£20800 | NA | 0.97 (0.82-1.15) | 1.23 (0.97-1.57) | 1.40 (1.07-1.83) |
|  | <£10400 | NA | 1.10 (0.92-1.32) | 1.68 (1.32-2.15) | 2.80 (2.14-3.64) |

Missing data in confounders (*n*=471): gestational age (*n*<10), maternal education (*n*=32), and disposable household income (*n*=445).

Missing data cognitive tests (based on the number of children participating at the oldest age at which the cognitive test was taken): Bracken School Readiness Assessment-Revised (*n*=1460), British Ability Scale II Naming Vocabulary at ages 3 and 5 (*n*=774), and British Ability Scale II Pattern Construction at ages 5 and 7 (*n*=313).

Odds ratios adjusted for all covariates listed in the table and sample weights.
