# Supplementary material for: Is amblyopia associated with school readiness and cognitive performance during early schooling? Findings from the Millennium Cohort Study
Source: PLoS One. 2020 Jun 19;15(6):e0234414. doi: 10.1371/journal.pone.0234414 (PMC7304573; doi:10.1371/journal.pone.0234414)
Supplement: S3 Table — a BAS II NV, British Ability Scale II Naming Vocabulary at ages 3 and 5; BAS II PC, British Ability Scale II Pattern Construction at ages 5 and 7. b Estimates adjusted for all covariates, sample weights, and random effect on child. (DOCX) [file pone.0234414.s003.docx]

Table S3: Associations with cognitive abilities and age-related trajectories using British Ability Scale II tests.

| Covariate | Category | BAS II NV  Estimate (se), *p*-value | BAS II PC Estimate (se), *p*-value |
| --- | --- | --- | --- |
| Baseline |  | 51.46 (0.32), <0.001 | 50.76 (0.44), <0.001 |
| Eye condition | No eye condition | (ref) | (ref) |
|  | Strabismus alone | -0.75 (0.81), 0.351 | -2.73 (1.04), 0.009 |
|  | Refractive amblyopia | -1.33 (0.72), 0.064 | -1.07 (0.98), 0.277 |
|  | Strabismic/mixed amblyopia | -3.21 (1.11), 0.004 | -2.67 (1.34), 0.046 |
| Treatment started  (time-variant) | No | (ref) | (ref) |
|  | Yes | -0.55 (0.70), 0.432 | 1.29 (0.88), 0.141 |
| Trajectory by age | Years | 2.03 (0.07), <0.001 | 0.88 (0.07), <0.001 |
| Sex | Girls | (ref) | (ref) |
|  | Boys | -5.60 (0.40), <0.001 | -4.55 (0.58), <0.001 |
| Ethnicity | White | (ref) | (ref) |
|  | Non-white | -7.45 (0.28), <0.001 | -2.75 (0.30), <0.001 |
| Birth order | 1 | (ref) | (ref) |
|  | 2 | -1.55 (0.18), <0.001 | 0.35 (0.20), 0.081 |
|  | 3+ | -3.29 (0.22), <0.001 | -0.37 (0.24), 0.121 |
| Gestational age | ≥37 weeks | (ref) | (ref) |
|  | <37 weeks | -0.66 (0.32), 0.039 | -1.29 (0.35), <0.001 |
| Maternal education | A-levels or higher | (ref) | (ref) |
|  | O-levels | -3.08 (0.18), <0.001 | -2.14 (0.20), <0.001 |
|  | None | -5.88 (0.28), <0.001 | -4.16 (0.30), <0.001 |
| Household language | English | (ref) | (ref) |
|  | Non-English | -5.93 (0.60), <0.001 | 0.48 (0.59), 0.415 |
| Household income | ≥£20800 | (ref) | (ref) |
|  | £10400-£20800 | -1.88 (0.20), <0.001 | -1.46 (0.21), <0.001 |
|  | <£10400 | -4.37 (0.22), <0.001 | -2.84 (0.24), <0.001 |
| Trajectory by age*sex | Girls | (ref) | (ref) |
|  | Boys | 1.03 (0.09), <0.001 | 0.58 (0.09), <0.001 |

BAS II NV, British Ability Scale II Naming Vocabulary at ages 3 and 5; BAS II PC, British Ability Scale II Pattern Construction at ages 5 and 7.

Estimates adjusted for all covariates, sample weights, and random effect on child.
