# Supplementary material for: Is amblyopia associated with school readiness and cognitive performance during early schooling? Findings from the Millennium Cohort Study
Source: PLoS One. 2020 Jun 19;15(6):e0234414. doi: 10.1371/journal.pone.0234414 (PMC7304573; doi:10.1371/journal.pone.0234414)
Supplement: S1 File — (DOCX) [file pone.0234414.s004.docx]

**Regression model assumptions**

The logistic model assumptions of linearity, independence of errors, and multicollinearity were checked by looking at interactions between predictor and its log transformation, Durbin-Watson tests, and variance inflation factors, respectively. The LME model’s assumptions of linearity, homeogeneity of variance, and normally distributed errors were checked by plots of predictor against studentised residuals, Levene’s tests, and QQ plots, respectively.
